# Ca^2+^-Dependent Glucose Transport in Skeletal Muscle by Diphlorethohydroxycarmalol, an Alga Phlorotannin: *In Vitro* and *In Vivo* Study

**DOI:** 10.1155/2021/8893679

**Published:** 2021-02-10

**Authors:** Hye-Won Yang, Yun-Fei Jiang, Hyo-Geun Lee, You-Jin Jeon, BoMi Ryu

**Affiliations:** ^1^Department of Marine Life Science, Jeju National University, Jeju 63243, Republic of Korea; ^2^Marine Science Institute, Jeju National University, Jeju 63333, Republic of Korea

## Abstract

Diphlorethohydroxycarmalol (DPHC), a type of phlorotannin isolated from the marine alga *Ishige okamurae*, reportedly alleviates impaired glucose tolerance. However, the molecular mechanisms of DPHC regulatory activity and by which it exerts potential beneficial effects on glucose transport into skeletal myotubes to control glucose homeostasis remain largely unexplored. The aim of this study was to evaluate the effect of DPHC on cytosolic Ca^2+^ levels and its correlation with blood glucose transport in skeletal myotubes *in vitro* and *in vivo*. Cytosolic Ca^2+^ levels upon DPHC treatment were evaluated in skeletal myotubes and zebrafish larvae by Ca^2+^ imaging using Fluo-4. We investigated the effect of DPHC on the blood glucose level and glucose transport pathway in a hyperglycemic zebrafish. DPHC was shown to control blood glucose levels by accelerating glucose transport; this effect was associated with elevated cytosolic Ca^2+^ levels in skeletal myotubes. Moreover, the increased cytosolic Ca^2+^ level caused by DPHC can facilitate the Glut4/AMPK pathways of the skeletal muscle in activating glucose metabolism, thereby regulating muscle contraction through the regulation of expression of troponin I/C, CaMKII, and ATP. Our findings provide insights into the mechanism of DPHC activity in skeletal myotubes, suggesting that increased cytosolic Ca^2+^ levels caused by DPHC can promote glucose transport into skeletal myotubes to modulate blood glucose levels, thus indicating the potential use of DPHC in the prevention of diabetes.

## 1. Introduction

Glucose is stored as glycogen in the skeletal muscle, a major site of glucose uptake that is critical to whole-body glucose metabolism in humans. Previous studies have shown that genetic activation of glycolytic metabolism in the skeletal muscle leads to an improvement in whole-body glucose homeostasis in mice [1]. Indeed, elevation in blood glucose levels results in increased glycolytic metabolism, which may represent an important aspect of the adaptive response in skeletal muscle [1].

Glucose uptake to control the blood glucose homeostasis can be induced through the activation of muscle contraction [2]. Some of the signaling mechanisms that mediate an increase in the expression of glucose transporter 4 (Glut4) in response to exercise have been identified to be driven by a rise in cytosolic Ca^2+^ levels due to increased release of Ca^2+^ from the sarcoplasmic reticulum (SR) [3, 4]. These studies showed that an increase in glucose uptake during muscle contractions does not require membrane depolarization but only a release of Ca^2+^ to the cytoplasm [5]. Cytosolic Ca^2+^ in the skeletal muscle regulates several signaling pathways and metabolic events related to contraction and relaxation [6].

Increased cytosolic Ca^2+^ levels in the skeletal muscle leads to the initiation of contraction, generating signals for the activation of Glut4 translocation [7, 8]. In addition, contractile activity results in an increase in the phosphorylation of Ca^2+^/calmodulin-dependent protein kinase II (CaMKII) in skeletal muscle, leading to the upregulation of Glut4 [4]. Glucose uptake in the skeletal muscle tissue is achieved by at least two major mechanisms: (1) insulin-dependent activation of PI3-K/Akt and (2) activation of AMPK by muscle contraction due to exercise, in order to maintain energy balance [9]. Activation of AMPK plays a key role in maintaining cellular energy levels and stimulation of glucose uptake.

Furthermore, muscle contraction can stimulate Glut4 and 5′ AMP-activated protein kinase (AMPK) expression to increase adenosine triphosphate (ATP) production [10, 11]. ATP hydrolysis, via the interaction between actin and myosin molecules, generates the force for muscle contraction [12]. The actomyosin ATPase activity is regulated by troponin-tropomyosin interaction, which is a crucial part of the protein interaction [13]. In addition, the binding of Ca^2+^ to troponin results in the contraction of muscle fibers [14]. In particular, the Ca^2+^ regulation mechanisms include the release upon Ca^2+^ binding to troponin C and the inhibition of contractile interaction through troponin I.

Previous studies have shown that diabetes mellitus is commonly linked to a decreased efficiency in utilizing ATP as energy in the skeletal muscles [15]. Increasing glucose uptake is derived from muscle contractile activity, resulting in the prevention of diabetes [16]. Alterations in Ca^2+^ homeostasis in skeletal muscles are strongly associated with metabolic stress and diabetes, as Ca^2+^ is indispensable for glucose uptake during muscle contraction in response to fluctuations in blood glucose [17, 18]. Therefore, in this study, we hypothesized that glucose transport can be regulated by the Ca^2+^-dependent cytosolic signaling pathways in muscle cells; this helps in regulating blood glucose levels.

In our previous studies, we demonstrated that the marine alga *Ishige okamurae* shows antidiabetic associated responses through the modulation of blood glucose levels as well as inhibition of carbohydrate-digestive enzymes [19]. In addition, diphlorethohydroxycarmalol (DPHC), a phlorotannin isolated from *Ishige okamurae*, was revealed to have potential antidiabetic activity via prominent inhibitory effects against the *α*-glucosidase and *α*-amylase enzymes [20]. However, regulating glucose homeostasis and the underlying mechanisms of glucose uptake in skeletal muscle by DPHC treatment to control blood glucose levels have not yet been examined.

In the present study, we found that during impaired glucose tolerance, DPHC can induce glucose uptake in the skeletal muscle to improve blood glucose homeostasis. In addition, we explored the effects of DPHC on cytosolic Ca^2+^ levels in the skeletal muscle and the underlying signaling mechanisms in *in vitro* and *in vivo* models. Further, the effect of DPHC on muscle contraction was evaluated in a zebrafish model with alloxan-induced hyperglycemia. Data from this study demonstrate that glucose transport is an important part of the mechanism underlying the effect of DPHC and further provide insights into strategies aimed at improving glucose homeostasis to help ameliorate metabolic disorders in patients with diabetes in the future.

## 2. Materials and Methods

### 2.1. Materials

Diphlorethohydroxycarmalol (DPHC) was isolated as previously described by Heo et al. [19]. Fluo-4 Direct™ Calcium Assay kit was purchased from Invitrogen by Thermo Fisher Scientific (Waltham, MA, USA). 3-(4,5-Dimethylthiazol-2-yl)-2,5-diphenylte-trazolium bromide (MTT); 2-deoxy-2-[(7-nitro-2,1,3-benzoxadiazol-4-yl)amino]-D-glucose (2-NBDG); 1,2-bis(2-aminophenoxy)ethane-N,N,N′,N′-tetraacetic acid tetrakis(acetoxymethyl ester) (BAPTA-AM); D-(+)-glucose (glucose); 2,4,5,6(1H,3H)-pyrimidinetetrone (alloxan monohydrate); and 1,1-dimethylbiguanide hydrochloride (metformin) were purchased from Sigma-Aldrich (St. Louis, MO, USA). Antibodies against phospho-AMP-activated protein kinase (Thr172), AMPK and p-Akt (Ser473), Akt, Glut4, and GAPDH were obtained from Cell Signaling Technology (Bedford, MA, USA), and anti-rabbit secondary antibodies were purchased from Santa Cruz Biotechnology (Santa Cruz, CA, USA). ATP Assay Kit (colorimetric/fluorometric) was purchased from Abcam (Abcam Inc., Cambridge, USA).

### 2.2. C2C12 Cell Culture, Differentiation, and Cell Viability Assay

Previous studies have shown that C2C12 cells represent a model to study insulin resistance in diabetes and improvement in muscle function [21, 22]. C2C12 skeletal muscle cells (American Type Culture Collection) were maintained in DMEM with 10% FBS and antibiotics, at 37°C in a humidified incubator of 5% CO_2_. When the cells reached 80% confluence, C2C12 cells were differentiated into skeletal myotubes in DMEM-low glucose with 2% horse serum for 5 days. Viability levels of C2C12 cells were determined by the ability of mitochondria to convert MTT to an insoluble formazan product. Cells were maintained in 96-well plates at a density of 3 × 10^4^ cells/well and subsequently subjected to different concentrations of DPHC (0.1, 2, 10, and 30 *μ*M) for 24 h. Cells were pretreated with MTT solution (2 mg/mL) for 3 h. Subsequently, cell density was determined by measuring optical density (OD) at 540 nm using a microplate reader (Gen5 version 2.05, BioTek, Winooski, Vermont, USA).

### 2.3. Glucose Uptake Assay

Differentiated cells and skeletal myotubes were maintained in serum-free and low-glucose DMEM for 6 h before treatment with DPHC. After incubation, cells were treated with DPHC in glucose-free media. Subsequently, 2-NBDG at 50 *μ*M final concentration was added for 24 h at 37°C. Then, cells were washed twice with PBS, serum-free medium was added, and the fluorescence intensity was immediately measured in a microplate reader at an excitation wavelength of 485 nm and an emission wavelength of 530 nm. After being taken up by the cells, 2-NBDG was converted into a nonfluorescent derivative (2-NBDG metabolite). The overall glucose uptake was obtained by quantifying the diminution in the fluorescence. The assay was performed as described by Blodgett et al. [23].

### 2.4. Cytosolic Ca^2+^ Level by Fluo-4 in Skeletal Myotubes

Cytosolic Ca^2+^ level was detected using the Ca^2+^-sensitive probe Fluo-4 in 1x PBS (phosphate-buffered saline, comprised of 140 mM NaCl, 5.9 mM KCl, 1.8 mM MgCl_2_, 10 mM HEPES, 11.5 mM glucose, 1.2 mM NaH_2_PO_4_, and 5 mM NaHCO_3_). Skeletal myotubes were incubated in 1x Fluo-4 for 30 minutes at 37°C, washed with PBS and added 1x PSS with 0.1% BSA. After recording basal fluorescence for 10 sec at interval time of 1 sec, cells were directly added 1x PSS or DPHC (3, 10, and 30 *μ*M) in 0.1% BSA. After recording fluorescence for 55 sec with interval time of 1 sec, reflecting cytosolic Ca^2+^ levels were obtained to the microscope (Gen 5 version 3.03, BioTek, Winooski, Vermont, USA). A box plot was used to visualize descriptive statistics of the different groups [24].

### 2.5. Experimental Animals

#### 2.5.1. Maintenance of Parental Zebrafish and Collection of Embryos

Adult zebrafish was obtained from a commercial dealer (Jeju aquarium, Jeju, Korea). Fifteen fishes were kept in 3.5 L acrylic tank according to the conditions: 28.5 ± 1°C, fed two times a day (Tetra GmgH D-49304, Melle, Germany) with a 14/10 light/dark cycle [25]. Embryos were mated, and spawning was stimulated by setting of light, after breeding 1 female and 2 males. Embryos were collected within 30 min and transferred to Petri dishes containing embryo media. The zebrafish experiment received approval from the Animal Care and Use Committee of the Jeju National University (Approval No. 2017-0001).

#### 2.5.2. Toxicity of DPHC in Zebrafish Embryos

From approximately 4 hours postfertilization (hpf), embryos (*n* = 15) were transferred to individual wells of 12-well plates containing 950 *μ*L embryo media with 50 *μ*L of DPHC (0.1, 2, 10, and 30 *μ*M). Percent survival of zebrafish embryos exposed to DPHC up to 168 hpf was measured.

#### 2.5.3. Cytosolic Ca^2+^ Level Measurement in Zebrafish Larvae

In accordance with a previous study [26], the cytosolic Ca^2+^ level of zebrafish larvae was detected using the Ca^2+^-sensitive probe, Fluo-4. Zebrafish larvae were incubated in the 2x Fluo-4 for 30 minutes at 28.5 ± 1°C and subsequently treated with DPHC (0.1, 2, and 10 *μ*M). As a control, to ensure if the Ca^2+^ signal was completely blocked by chelation of cytosolic Ca^2+^ with BAPTA-AM, we preincubated fish with 0.1 mM BAPTA-AM for 1 h before loading the Fluo-4.

#### 2.5.4. Blood Glucose Level

Wild-type zebrafish were exposed to 2 mg/mL alloxan for 1 h and transferred to 1% glucose for another 1 h. The media was then changed to water for 1 h, and zebrafish were injected with saline, or DPHC, or metformin, with or without BAPTA-AM for 90 min. Adult zebrafish were divided into six groups: normal, alloxan-treated group, DPHC (0.3 *μ*g/g body weight), metformin (5 *μ*g/g body weight; a positive control with known blood glucose level-controlling activity) [27], BAPTA-AM (3 *μ*g/g body weight), and BAPTA-AM (3 *μ*g/g body weight) with DPHC (0.3 *μ*g/g body weight). Blood glucose level was measured using the protocol described by Zang et al. [28].

#### 2.5.5. Plasma Membrane Protein Extraction of Zebrafish Muscle Tissue

In accordance with a previous study [29], membrane protein of zebrafish muscle tissue was extracted using a membrane protein extraction kit (Catalog number 89842, Thermo Scientific, Waltham, Massachusetts, USA) according to the manufacturer's protocols. For the zebrafish muscle tissue collection, the fish's head, tail, fins, skin, and internal organs were removed using a scalpel and forceps. Zebrafish muscle tissue (20-40 mg) was washed in 4 mL of cell wash solution and homogenized in 1 mL of permeabilization buffer. And then, tissue was incubated for 10 min at 4°C. The homogenates were centrifuged at 16,000 × g for 15 min at 4°C to pellet permeabilized cells, and then the supernatant containing cytosolic proteins was removed. The membrane proteins pellet was resuspended in 1 mL of solubilization buffer, then pipetted up and down, incubated 30 min at 4°C, and centrifuged at 16,000 × g for 15 min at 4°C. The supernatant containing solubilized membrane and membrane-associated proteins was stored for western blot analysis.

### 2.6. Western Blot Analysis

Western blot analysis was carried out using the protocol described by Ko et al. [30]. The phospho-AMPK (Thr172), total-AMPK (Ser473), phosphor-Akt, total-Akt, and Glut4 were used at 1 : 1000 dilution, and secondary antibodies were used at 1 : 3000 dilution.

### 2.7. ATP Assay

Using 0.1 mM ATP standard dilution, a standard curve was generated following the ATP assay kit protocol (ab83355, Abcam, America). 10 mg of muscle tissue was harvested for each assay and washed in cold PBS. Tissue was homogenized in ice-cold 2 N PCA with 10-15 passes. Following maintaining on ice for 30-45 min, samples were centrifuged at 13,000 g for 3 min at 4°C, and supernatants were collected. Samples were adjusted to pH between 6.5 and 8 to neutralize the samples and precipitate the excess PCA. Samples were centrifuged at 13,000 g for 15 min at 4°C, and supernatants were collected. The supernatants were transferred to individual wells of 96-well plates mixed with the same volume of ATP reaction mix and incubated at 21 ± 1°C for 30 min, protected from light. ATP levels in each sample were determined by measuring optical density (OD) at 570 nm using a microplate reader (Gen5 version 2.05, BioTek, Winooski, Vermont, USA). The amount of ATP in the samples was calculated according to the standard curve.

### 2.8. Immunofluorescence (IF)

To conduct histological analysis, the muscle tissue of zebrafish was fixed in Bouin's solution for 24 h and subsequently transferred to 70% ethanol for storage. Following this, the tissue was dehydrated in a graded ethanol series and embedded in paraffin. The muscle tissue was sectioned into 7 *μ*m sections using a microtome (Leica, Nussloch, Germany) and mounted onto glass slides. The tissue slides were deparaffinized and treated with animal serum to block antibody binding and then incubated with anti-GLUT4 antibodies (1 : 400), troponin I (1 : 400), and troponin C (1 : 400) overnight at 4°C. In addition, the tissue slides were rinsed with PBS and incubated with fluorescent-dye conjugated secondary antibodies (1 : 200) for 1 h at 21 ± 1°C. Rinsed tissue slides were mounted with slide mounting medium (SIGMA). The fluorescence from tissue slides was observed and imaged under the microscope (Gen5 version 3.03, BioTek, Winooski, Vermont, USA).

### 2.9. Statistical Analysis

All of the data were presented as means ± standard error (SE). The mean values were calculated based on data from at least three independent experiments which were conducted on separate days using freshly prepared reagents. All the experiments were statistically analyzed using one-way analysis of variance (one-way ANOVA) and Fisher's LSD test (in GraphPad Prism Version 7.03). A *p* value of less than 0.05 (^#^*p* < 0.05, ^##^*p* < 0.01, ^###^*p* < 0.001, and ^####^*p* < 0.0001) was considered statistically significant and compared with the nontreated group. A *p* value of less than 0.05 (^∗^*p* < 0.05, ^∗∗^*p* < 0.01, ^∗∗∗^*p* < 0.001, and ^∗∗∗∗^*p* < 0.0001) was considered statistically significant and compared with the no sample-treated group.

## 3. Results

### 3.1. Regulation of Blood Glucose Levels in Adult Zebrafish

**Figure 1:**
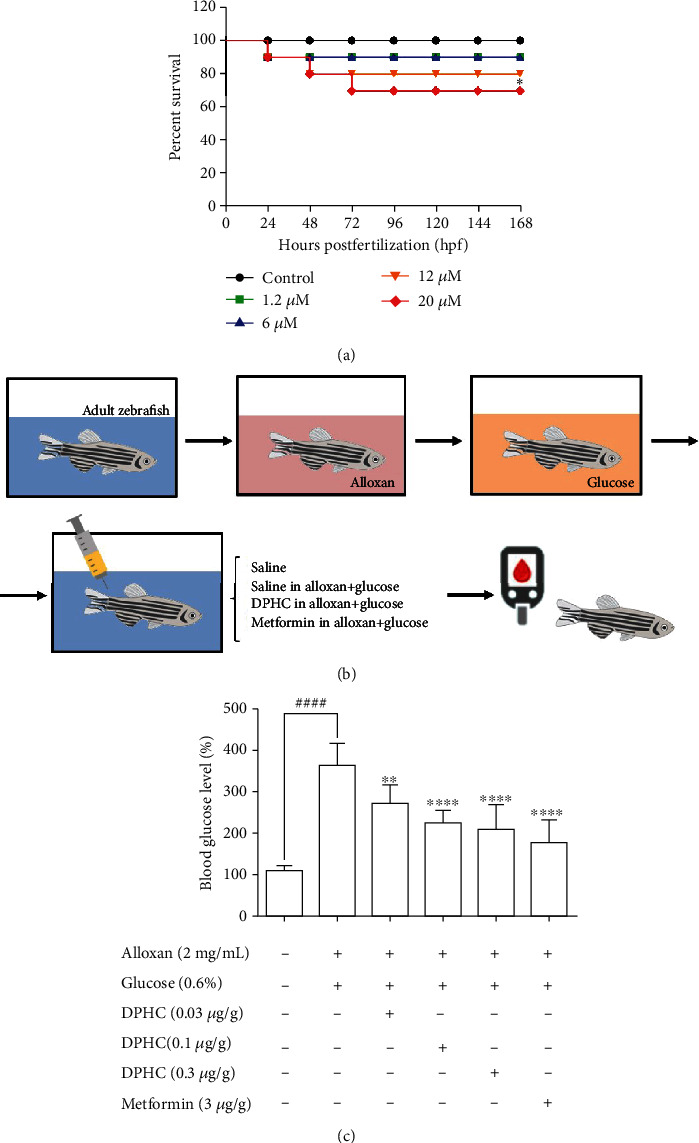
Evaluation of toxicity of DPHC by assessment of survival rate and measurement of blood glucose level in zebrafish. The zebrafish were treated with various concentrations of DPHC from *Ishige okamurae*. (a) Survival curves of zebrafish embryos after exposure to DPHC (*n* = 15 for each group). (b) Diagram and (c) result for blood glucose level of zebrafish injected with DPHC and metformin for 90 min. Data are expressed as the mean ± SE (*n* = 3 for each group). ^∗^^,#^Values having different superscripts are significantly different at ^∗^*p* < 0.05, ^∗∗^*p* < 0.01, and ^∗∗∗∗^*p* < 0.0001 compared with the no sample-treated group; ^####^*p* < 0.0001 compared with the nontreated group.

To assess whether blood glucose levels are influenced by DPHC, we used an alloxan-induced hyperglycemic zebrafish model [25]. Initially, to evaluate the toxicity of DPHC, zebrafish embryos were exposed to 0.1, 2, 10, and 30 *μ*M of DPHC in the embryo media for 168 hpf. As shown in Figure 1(a), 90%, 90%, and 80% of the zebrafish survived after treatment with 1.2, 6, and 12 *μ*M DPHC, respectively, whereas 30 *μ*M DPHC treatment showed a significant decrease in survival to 70 ± 4.71% compared to that observed in the control group. Therefore, the subsequent zebrafish experiments were conducted with concentrations that permitted ≥80% survival (Figure 1(a)).

Zebrafish treated with 2 mg/mL alloxan showed pancreatic islet damage, leading to reduced glucose-mediated insulin secretion, resulting in a diabetic condition (Figure 1(b)). Blood glucose levels of zebrafish in the 0.6% glucose group increased 3.28-fold compared to those in the normal group (Figure 1(c)), which were comparable to the blood glucose levels in a hyperglycemic mouse model. Injection with increasing concentrations of DPHC (0.03, 0.1, and 0.3 *μ*g/g) in 0.6% glucose to zebrafish decreased blood glucose levels by 269, 223, and 206 mg/dL, respectively, whereas metformin (3 *μ*g/g) led to a 176 mg/dL decrease in the blood glucose level. These data suggest that DPHC can reduce blood glucose levels in a hyperglycemic zebrafish model.

### 3.2. Measurement of Cell Viability and Glucose Uptake in Skeletal Myotubes

**Figure 2:**
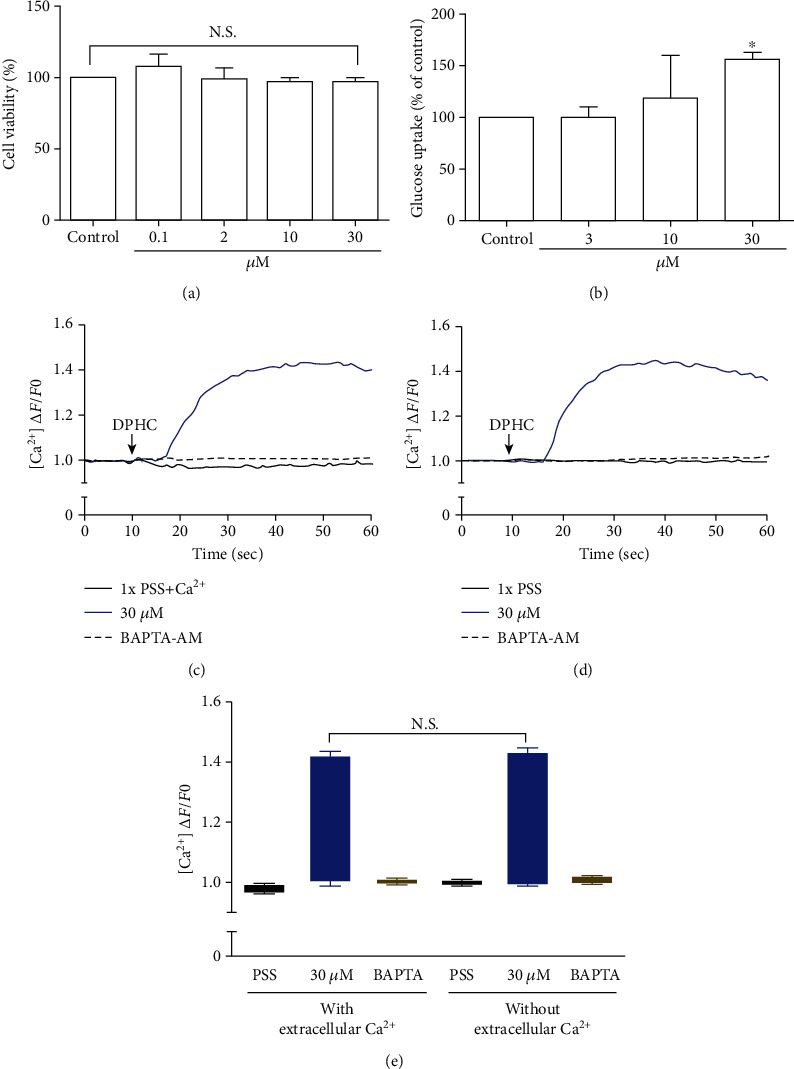
Determination of cell viability by the MTT assay in the myotubes. (a) Myotubes were incubated with the indicated concentrations of DPHC for 24 h. (b) Myotubes were starved in serum-free media and incubated for 24 h with DPHC (3, 10, and 30 *μ*M) and 2-NBDG (50 *μ*M). Detection of cytosolic Ca^2+^ levels using the Fluo-4 calcium indicator in the myotubes. Myotubes were loaded with Fluo-4 in PSS in the (c) presence or (d) absence of Ca^2+^ and treated with control (PSS only), 30 *μ*M of DPHC, or BAPTA-AM. Box plots representation of (e) the cytosolic Ca^2+^ levels in myotube responses after 30 *μ*M of DPHC or BAPTA-AM treatment as presented in (c) and (d). Data are expressed as the mean ± SE, *n* = 3 per group, ^∗^*p* < 0.05 compared with the nontreated group. N.S.: no significance compared with the DPHC in the presence or absence of extracellular Ca^2+^.

As glucose uptake by skeletal muscles is the key step in glucose homeostasis [31], we examined the effect of DPHC on glucose uptake in differentiated myocytes. Initially, the cells were treated with DPHC (0.1, 2, 10, and 30 *μ*M) for 24 h, and cell viability was assessed using an MTT assay. As shown in Figure 2(a), cells treated with DPHC did not show a significant difference in cell viability compared to those of the control group. It was therefore confirmed that these concentrations of DPHC were nontoxic and were used for further *in vitro* experiments.

Glucose uptake following DPHC treatment in skeletal myotubes was evaluated without insulin stimulation (Figure 2(b)). Treatment of the skeletal myotubes with DPHC led to an increase in glucose uptake in a concentration-dependent manner, and 30 *μ*M of DPHC led to a significant increase in glucose uptake of 154% compared to that in the control group. These data suggested that DPHC treatment can induce glucose uptake in the absence of insulin in skeletal myotubes.

### 3.3. Increasing the Cytosolic Ca^2+^ Level in Skeletal Myotubes

To investigate the change of cytosolic Ca^2+^ levels by DPHC treatment, we performed Ca^2+^ imaging using Fluo-4-AM, a cell permeable and sensitive calcium indicator. Exposure of skeletal myotubes to DPHC in the presence of extracellular Ca^2+^ in this PSS buffer with the addition of CaCl_2_ resulted in a rapid and robust increase in cytosolic calcium levels (Figure 2(c)). Similarly, in the absence of external Ca^2+^, elevation in cytosolic Ca^2+^ levels upon DPHC treatment in the skeletal myotubes was also observed, indicating that no difference was observed by the presence or absence of extracellular Ca^2+^ (Figure 2(d)). In Figure 2(e), the box plot of the Ca^2+^ response for 60 sec from Figures 2(c) and 2(d) in the presence or absence of extracellular Ca^2+^ shows that cytosolic Ca^2+^ levels following 30 *μ*M DPHC treatment were not significantly altered by extracellular Ca^2+^. Additionally, the skeletal myotubes pretreated with BAPTA-AM, a cytosolic Ca^2+^ chelator, elicited no change in cytosolic Ca^2+^ upon DPHC treatment in both the presence or absence of extracellular Ca^2+^. Elevation of cytosolic Ca^2+^ in skeletal myotubes treated with DPHC regardless of the existence of extracellular Ca^2+^ suggests that the Ca^2+^ increase by DPHC is likely to be related to the release of Ca^2+^ to the cytosol from the SR and not to Ca^2+^ influx by plasma membrane Ca^2+^ channels.

**Figure 3:**
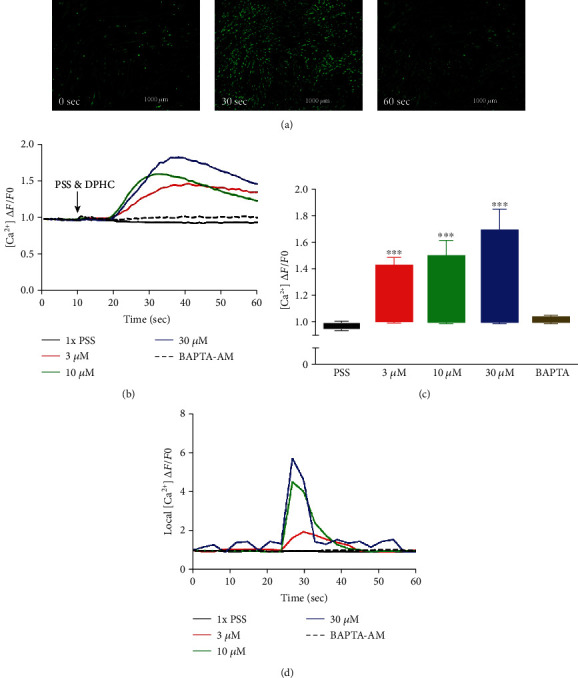
Detection of cytosolic Ca^2+^ levels in the absence of extracellular Ca^2+^ using the Fluo-4 indicator in the myotubes. (a) Representative images of myotubes at time zero (0 sec) and after stimulation with 30 *μ*M of DPHC at time 30 sec and 60 sec. (b) Traces and (c) box plot representation of Ca^2+^ levels in response to addition of DPHC or BAPTA-AM in myotubes. The fluorescence levels of single myotubes after addition of DPHC or BAPTA-AM was also monitored (d). ^∗^*p* < 0.05 and ^∗∗∗^*p* < 0.001 compared with the PSS group.

Next, the level of cytosolic Ca^2+^ with different concentrations of DPHC (3, 10, and 30 *μ*M) was analyzed by measuring fluorescence changes in skeletal myotubes in the absence of extracellular Ca^2+^. Cytosolic Ca^2+^ was instantly observed to increase upon DPHC treatment, while in the presence of BAPTA-AM, this calcium response was abolished (Figures 3(a) and 3(b)). The Ca^2+^ response in total skeletal myotubes upon DPHC treatment was quantified as shown in Figure 3(c), indicating that cytosolic Ca^2+^ significantly increased with 3, 10, and 30 *μ*M DPHC compared to that with PSS treatment. This response was also observed in single skeletal myotubes (Figure 3(d)). These data showed that the Ca^2+^ release from the SR is induced in response to DPHC treatment of the skeletal myotubes.

### 3.4. DPHC Engages Cytosolic Ca^2+^ Signaling to Regulate Blood Glucose

**Figure 4:**
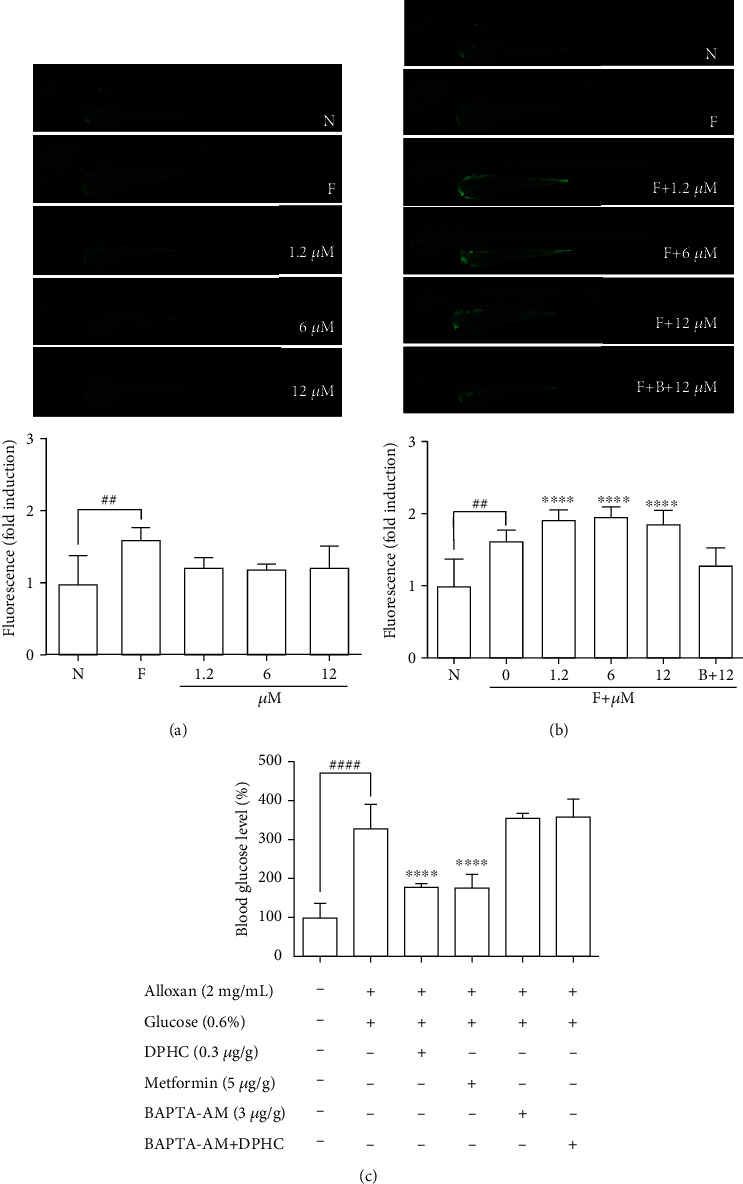
Detection of cytosolic Ca^2+^ changes using the Fluo-4 in zebrafish larvae. (a) Zebrafish larvae were stimulated with only DPHC (1.2, 6, and 12 *μ*M). (b) Zebrafish larvae were stimulated with DPHC in Fluo-4 and compared with/without Fluo-4 groups. Changes in cytosolic Ca^2+^ levels were measured by changes in fluorescence intensity of Fluo-4 using ImageJ. (c) Measurement of blood glucose level in zebrafish. Zebrafish were injected with BAPTA-AM (3 *μ*g/g body weight) for 1 h, after which the zebrafish were injected with DPHC (0.3 *μ*g/g body weight) for 90 min. Experiments were performed in triplicate, and the data are expressed as the mean ± SE, *n* = 4 per group. ^∗^^,#^Values having different superscripts are significantly different at ^∗∗∗∗^*p* < 0.0001 compared with the no sample-treated group; ^##^*p* < 0.01 and ^####^*p* < 0.0001 compared with the nontreated group.

Given that cytosolic Ca^2+^ release is indispensable for glucose uptake in myocytes [32], we postulated that DPHC might engage Ca^2+^ signaling to regulate skeletal muscle glucose homeostasis. To test this, we performed Ca^2+^ imaging studies in 3 hdf zebrafish embryos using Fluo-4 dye in the absence of external Ca^2+^ following exposure to DPHC. Initially, we confirmed that no significant fluorescence was induced by treatment with different concentrations of DPHC alone (without Fluo-4) compared to that in the controls (N, no Fluo-4 and no drugs exposed, and F, only Fluo-4 exposed) (Figure 4(a)). Moreover, fluorescence in the zebrafish in the Fluo-4 alone (F) sample increased 1.62-folds compared to that in the normal (N) control. These data showed that the intracellular Ca^2+^ in zebrafish embryos were detected by Fluo-4. Next, the effect of DPHC in Fluo-4-exposed embryos was assessed. Zebrafish embryos exposed to DPHC and Fluo-4 had a rapid rise in cytosolic Ca^2+^; however, this increase was abolished with preincubation of the 10 *μ*M DPHC-treated zebrafish embryos with 0.1 mM BAPTA-AM for 1 h (Figure 4(b)). This finding suggests that DPHC can induce cytosolic Ca^2+^ elevation in zebrafish embryos.

To examine whether Ca^2+^ is required for the blood glucose-lowering effect of DPHC, we treated alloxan-induced hyperglycemic zebrafish with DPHC and measured blood glucose and ATP levels. As shown in Figure 4(c), the alloxan-treated group had blood glucose levels of up to 278 mg/dL, while they were significantly lower in the DPHC (0.3 *μ*g/g) and metformin (3 *μ*g/g) (149 mg/dL and 151 mg/dL, respectively) treatment groups and the blank group (first column, saline only injected group). Importantly, this blood glucose-lowering effect of DPHC was significantly attenuated with BAPTA-AM treatment, resulting in blood glucose being at the same level as in the non-DPHC-treated zebrafish. Furthermore, STZ/saline groups in the diabetic mouse model showed a lower glucose tolerance, with higher glucose level after 90 min, compared with the normal/saline group in Figure S1a. We calculated the area under the curve (AUC) of IGTT in each group. In Figure S1b, the AUC of IGTT in the STZ/saline group was significantly increased compared with that in the normal/saline group. However, the increased AUC of IGTT were significantly decreased by STZ/DPHC and STZ/metformin. In particular, the STZ/BAPTA+DPHC group showed no significant change in AUC of IGTT.

These results showed that the cytosolic Ca^2+^ release from the SR by DPHC is indispensable for its effect on blood glucose homeostasis in hyperglycemic zebrafish with suppressed insulin secretion, suggesting that DPHC-induced blood glucose homeostasis requires activation of cytosolic Ca^2+^ release and the downstream signaling pathway.

### 3.5. Assessment of the Expression of Glucose Transport Pathway Components in Zebrafish Muscle Tissue

**Figure 5:**
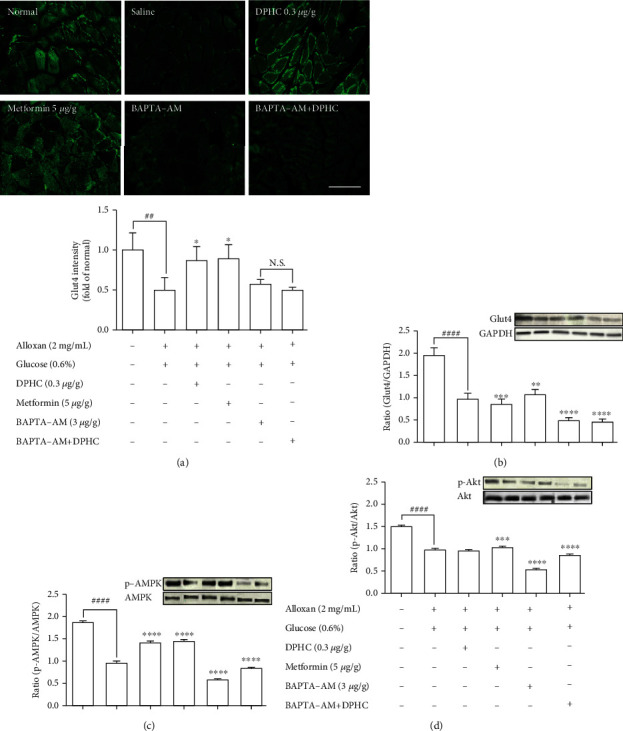
Expression of (a) Glut4 in zebrafish muscle tissues by immunofluorescence. Analysis of (b) Glut4, (c) AMPK, and (d) Akt signaling pathway in zebrafish muscle tissues by western blotting. The muscle extract was analyzed by western bottling, and the signal intensities were examined by the Fusion FX7 acquisition system (Vilber Lourmat, Eberhardzell, Germany). Experiments were performed in triplicate, and the data are expressed as mean ± SE, *n* = 4 per group. ^∗^^,#^Values having different superscripts are significantly different at ^∗^*p* < 0.05, ^∗∗^*p* < 0.01, ^∗∗∗^*p* < 0.001, and ^∗∗∗∗^*p* < 0.0001 compared with the no sample-treated group; ^##^*p* < 0.01 and ^####^*p* < 0.0001 compared with the nontreated group. N.S.: no significance compared with the BAPTA-AM group.

We examined the effect of DPHC on Glut4 translocation in the muscle membranes of hyperglycemic zebrafish by immunofluorescence. As shown in Figure 5(a), Glut4 intensity significantly decreased 0.5-fold relative to those in the nontreated group. When injected with DPHC or metformin, the Glut4 intensity increased to 0.87- or 0.90-fold relative to that in the alloxan-treated group. In particular, BAPTA-AM-injected groups with/without DPHC showed no significant change in intensity. This finding suggests that DPHC can induce Glut4 translocation by increasing the cytosolic Ca^2+^ level in hyperglycemic zebrafish muscle.

To evaluate the molecular components that are involved in the regulation of glucose uptake by DPHC, the protein levels of membrane-associated Glut4 and phosphorylation of AMPK and Akt in the muscle of alloxan-induced hyperglycemic zebrafish were analyzed by western blotting. As shown in Figures 5(b)–5(d), the membrane localization of Glut4 and phosphorylation levels of AMPK and Akt significantly decreased in the muscle of alloxan-induced hyperglycemic zebrafish compared to those in the blank-treated animals. However, DPHC and metformin promoted Glut4 translocation to the membrane and phosphorylation of AMPK, indicating that DPHC can stimulate the glucose transport pathway in hyperglycemic zebrafish. However, DPHC did not affect the expression of Akt compared to that in the alloxan-induced hyperglycemic zebrafish. As shown Figure S2, membrane Glut4 level of the STZ/saline group in the diabetic mouse model was significantly decreased compared with that in the normal/saline group. However, the decreased membrane Glut4 levels were significantly increased by STZ/DPHC and STZ/metformin. In particular, the STZ/BAPTA+DPHC group showed no significant change in membrane Glut4 level.

In contrast, Ca^2+^ depletion by BAPTA-AM failed to activate phosphorylation of these proteins, and protein levels remained unchanged even with DPHC treatment compared to those in the alloxan-induced hyperglycemic zebrafish, suggesting that DPHC can induce Ca^2+^-dependent activation of the Glut4/AMPK pathway in the muscles of hyperglycemic zebrafish to control glucose homeostasis.

### 3.6. Determination of Muscle Contraction in Zebrafish Muscle

**Figure 6:**
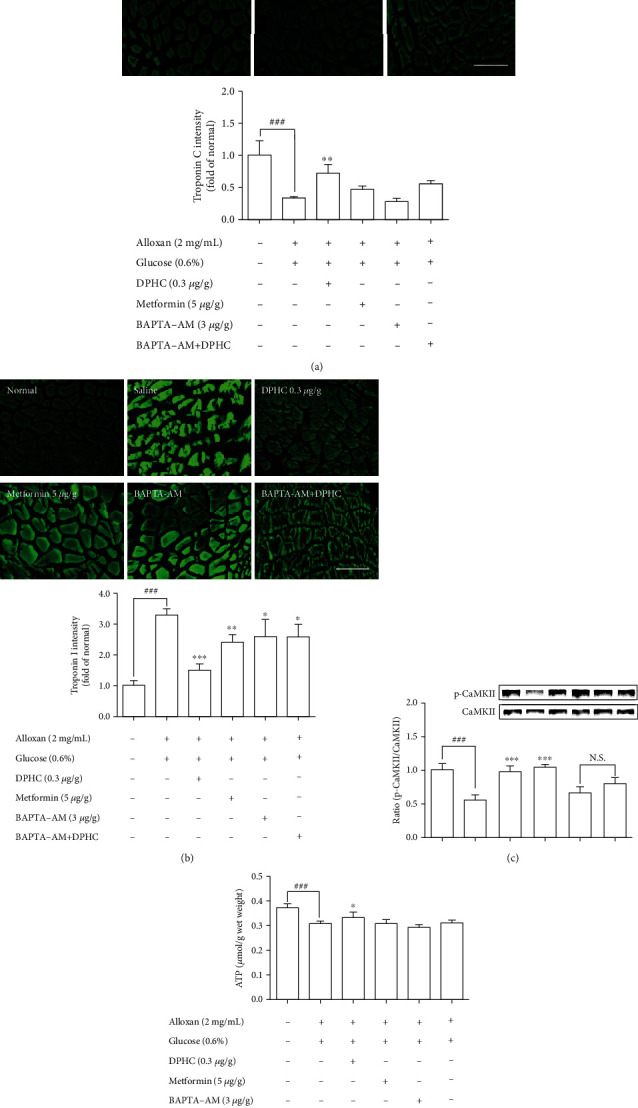
Measurement of (a) troponin C, (b) troponin I, (c) CaMKII, and (d) ATP levels in zebrafish muscle tissues. Zebrafish were injected with BAPTA-AM (3 *μ*g/g body weight) for 1 h, after which the zebrafish were injected with DPHC (0.3 *μ*g/g body weight) for 90 min. Experiments were performed in triplicate, and the data are expressed as mean ± SE, *n* = 3 per group. ^∗^^,#^Values having different superscript are significantly different at ^∗^*p* < 0.05, ^∗∗^*p* < 0.01, and ^∗∗∗^*p* < 0.001 compared with the no sample-treated group; ^###^*p* < 0.001 compared with the nontreated group.

To assess whether skeletal muscle contraction in hyperglycemic zebrafish is affected by DPHC, we examined troponin C and I intensity in hyperglycemic zebrafish using immunofluorescence. As shown in Figure 6(a), troponin C intensity in skeletal muscles of hyperglycemic zebrafish significantly decreased by 0.33-fold of that in the nontreated group (*p* < 0.001). However, DPHC significantly increased troponin C intensity (*p* < 0.01). In addition, troponin I intensity in the skeletal muscle of hyperglycemic zebrafish significantly increased to 3.3-fold of that in the nontreated group (Figure 6(b)). However, DPHC treatment significantly decreased troponin I intensity. These results indicated that DPHC can stimulate muscle contraction in hyperglycemic zebrafish.

We examined the CaMKIIs that are involved in the improvement of glucose homeostasis by DPHC in skeletal muscles of hyperglycemic zebrafish. As shown in Figure 6(c), DPHC treatment significantly reduced the CaMKII level in the alloxan-induced hyperglycemic zebrafish. However, BAPTA-AM-injected groups with/without DPHC did not significantly change the CaMKII expression level. This finding suggests that DPHC can induce muscle contraction by CaMKII expression, the latter being induced in turn by increased cytosolic Ca^2+^ level in the hyperglycemic zebrafish muscle. Furthermore, DPHC treatment modestly increased the alloxan-induced reduction in ATP levels in the skeletal muscle in hyperglycemic zebrafish, whilst this increase was reversed with BAPTA-AM treatment (Figure 6(d)). The data suggest that DPHC can improve glucose homeostasis by inducing muscle contraction in hyperglycemic zebrafish.

## 4. Discussion

Abnormal metabolism of glucose in diabetes reduces insulin sensitivity and induces postprandial hyperglycemia contractile activity in the skeletal muscle. Previous studies have reported the antidiabetic effects of *Ishige okamurae* extract on blood glucose level and insulin resistance in C57BL/KsJ-*db/db* mice [20]. We showed that DPHC isolated from *Ishige okamurae* significantly reduced the blood glucose level in the hyperglycemic zebrafish model with impaired glucose tolerance. In addition, DPHC can induce glucose uptake in the absence of insulin in C2C12 cells (Figure 2(b)). Our data suggest that DPHC can induce glucose transport in the skeletal muscle through contractile activity, resulting in the regulation of blood glucose level in a hyperglycemic model.

Glucose uptake by the skeletal muscle is regulated by glucose delivery to the skeletal muscle cells, surface membrane permeability to glucose, and flux through intracellular metabolism. In addition, the skeletal muscle possesses contractile activity that can effectively restore glucose regulation in an insulin-independent manner [2, 33]. Previous studies using different methods and models show that muscle contractions enhance glucose uptake similar to that by insulin action [33, 34]. Zebrafish models have been established to study a wide range of human pathologies, including genetic disorders and acquired diseases [35, 36]. Previous studies used alloxan or streptozotocin or only glucose water to generate an acute hyperglycemic zebrafish model [37, 38]. Shin et al. [38] suggested that a hyperglycemic zebrafish model is appropriate for experiments that call for a short-term model.

Skeletal muscle contraction is initiated by depolarization of the plasma membrane and T tubules, which not only triggers Ca^2+^ release from the SR but also leads to the interaction of actin and myosin filaments and development of tension in the fibers. The contraction and relaxation cycle produced by muscle fibers is called twitch [39]. In previous studies, the time course of Ca^2+^ in skeletal muscle fibers was studied by Ca^2+^ indicators [40], and Garcia and Schneider [41] also suggested that the kinetic properties that were characterized in the fast-twitch skeletal muscle fiber were controlled by Ca^2+^ release from the SR. The transient increase in calcium levels is important for intermediate signaling between the excitation and contraction phase in skeletal myotubes [42]. In this study, we present data supporting an association between the cytosolic Ca^2+^ release by DPHC in skeletal myotubes and its antidiabetes activity [20] that can improve impaired glucose uptake in diabetes to enable the maintenance of glucose homeostasis. Glucose uptake through the cell membrane is the rate-limiting step in glucose homeostasis in the skeletal muscle under physiological conditions [43] and is independently stimulated by Ca^2+^/contraction-dependent processes [44, 45]. The period between muscle stimulation and contraction is called latent period [46], which is a period of 10 seconds after DPHC treatment. After 10 seconds, the skeletal myotubes undergo a contracting period followed by a relaxation period. The basic unit of activity in the muscle fiber is the twitch that can induce short contractions [42]. DPHC significantly increased cytosolic Ca^2+^ levels in the skeletal myotubes, independent of extracellular Ca^2+^, ruling out the involvement of Ca^2+^ influx pathways and suggesting induction of Ca^2+^ release from the SR by DPHC [3, 5]. Although Ca^2+^ release from the SR of myotube cells can facilitate attachment/detachment of myosin to actin and the energy supply to ensure the excitation-contraction-relaxation cycle of muscle cells [47], the role of Ca^2+^ in glucose uptake, as well as its pathogenic contribution to diabetes, remains elusive.

The remarkable observation in our study is that cytosolic Ca^2+^ release by DPHC treatment can induce glucose uptake resulting in the modulation of blood glucose levels in the hyperglycemic zebrafish model, suggesting that cytosolic Ca^2+^ in skeletal myotubes can improve glucose homeostasis. Increased intracellular Ca^2+^ levels, even at low concentrations to induce contractions, provide the signal to activate Glut4 translocation to the cell surface in skeletal muscle independently of insulin [48]. Depending on the coordination and integration of several physiological systems, blood glucose homeostasis is maintained. Moreover, during exercise, an increased need for glucose by contracting muscle results in glucose uptake from blood into the working skeletal muscles [48, 49]. This study suggests that glucose homeostasis in muscles can be suggested as an important strategy for diabetes therapy [50] and can improve insulin sensitivity and reduce postprandial hyperglycemia [23, 30, 51].

Overexpression of key signaling proteins regulating energy metabolism in the skeletal muscle can improve metabolic disturbances associated with hyperglycemia. The diabetic mouse model generated using alloxan has been reported to affect expression of Glut4, resulting in a decreased uptake of glucose and increased blood glucose levels [52]. In addition, AMPK activation promotes Glut4 translocation and increases glucose uptake directly in the skeletal muscle [25]. Muscle contraction stimulates glucose transport by AMPK activation, increasing cytosolic Ca^2+^ level and Glut4 activation. Furthermore, previous studies have shown that increasing the cytosolic Ca^2+^ level induces glucose uptake in isolated rat muscle without increasing AMPK phosphorylation [34, 53, 54], suggesting that cytosolic Ca^2+^ levels increase glucose uptake during contractile activity. Our results showed that DPHC can stimulate Glut4 translocation and phosphorylation of AMPK, which was dependent on cytosolic Ca^2+^ in skeletal myotubes, while there was no effect on Akt activation. The cytosolic Ca^2+^ regulation by DPHC treatment in skeletal myotubes can reinforce the Glut4/AMPK pathways for a profound effect on glucose uptake metabolism. Our data further indicated that DPHC can normalize metabolic disturbances in diabetes and improve glucose homeostasis in skeletal muscles.

Skeletal muscle contraction is regulated by Ca^2+^ by controlling the action of specific regulatory proteins such as tropomyosin and troponin [55]. Troponin C is a Ca^2+^-binding component in muscle contraction. Troponin I is an inhibitory component of contractile interaction between myosin and actin in the presence of tropomyosin [56]. Our results showed that alloxan-induced hyperglycemic zebrafish can stimulate dysfunction of muscle contraction. We confirmed that the DPHC can promote muscle contraction by increasing cytosolic Ca^2+^ levels in the hyperglycemic zebrafish muscle. In addition, CaMKII activation promotes Glut4 translocation to increase glucose uptake directly in the skeletal muscle [4]. Previous studies have shown that caffeine treatment induces the activation of CaMKII by muscle contraction via Ca^2+^ release from the SR in rat muscle [53]. We confirmed that DPHC can stimulate CaMKII production in skeletal muscle tissues of hyperglycemic zebrafish. Our results showed that DPHC can enhance CaMKII expression levels; this is influenced by cytosolic Ca^2+^ in skeletal muscles of hyperglycemic zebrafish.

Muscle contraction initiates Ca^2+^ release into the myofibril and, as a result, increases ATP demand through the activity of myosin and Ca^2+^ ATPases. Since the SR (source of Ca^2+^) and mitochondria (source of ATP) are both necessary for the flawless execution of the contractile cycle [49], we confirmed that the ATP level in hyperglycemic zebrafish, the model where we observed the blood glucose-lowering effect of DPHC, was increased by DPHC treatment.

## 5. Conclusions

In this study, we uncovered the molecular mechanisms underlying the euglycemic effects of DPHC in the skeletal muscle of a hyperglycemic zebrafish model. DPHC can increase cytosolic Ca^2+^ level to activate Glut4/AMPK pathways of the skeletal muscle, which promotes glucose transport in hyperglycemic zebrafish. Also, increased Ca^2+^ by DPHC is linked with the regulation of troponin I/C, CaMKII, and ATP levels that enable muscle contraction. Therefore, the findings from this study provide a mechanistic and integrative approach demonstrating the upregulation of cytosolic Ca^2+^ by DPHC in the skeletal muscle as a potential therapeutic mechanism for improving glucose metabolism during diabetes.

## Data Availability

The data used to support the findings of this study are available from the corresponding authors upon request.

## Abbreviations

DPHC: Diphlorethohydroxycarmalol
SR: Sarcoplasmic reticulum
MTT: 3-(4,5-Dimethylthiazol-2-yl)-2,5-diphenylte-trazolium bromide
2-NBDG: 2-Deoxy-2-[(7-nitro-2,1,3-benzoxadiazol-4-yl)amino]-D-glucose
BAPTA-AM: 1,2-Bis(2-aminophenoxy)ethane-N,N,N′,N′-tetraacetic acid tetrakis(acetoxymethyl ester)
Glucose: D-(+)-Glucose
Alloxan: 2,4,5,6(1H,3H)-Pyrimidinetetrone
Metformin: 1,1-Dimethylbiguanide hydrochloride
AMPK: Phospho-AMP-activated protein kinase
PSS: Phosphate-buffered saline
Hpf: Hours postfertilization.

## Supplementary Materials

Supplementary Materials Figure S1: evaluation of IGTT in streptozotocin-induced diabetic mice. (a) Measurement of blood glucose level after glucose intake in mice fed with 30 mg/kg DPHC or 100 mg/kg metformin. Mice were injected with 2.5 mg/kg BAPTA-AM for 30 min, before glucose intake. (b) Quantitative analysis of the area under the curve (AUC) from IGTT. Data are expressed as the mean ± SE, *n* = 4 per group. ^∗^^,#^Values having different superscripts are significantly different at ^∗^*p* < 0.05 compared with the no sample-treated group; ^##^*p* < 0.01 compared with the nontreated group. Figure S2: expression of membrane and cytosolic Glut4 level in mice muscle tissues by western blotting. The muscle extract was analyzed by western bottling, and the signal intensities were examined by the Fusion FX7 acquisition system (Vilber Lourmat, Eberhardzell, Germany). Membrane Glut4 was normalized by cytosolic Glut4. Data are expressed as the mean ± SE, *n* = 4 per group. ^∗^^,#^Values having different superscripts are significantly different at ^∗^*p* < 0.05 and ^∗∗∗^*p* < 0.001 compared with the no sample-treated group; ^###^*p* < 0.001 compared with the nontreated group.
