# Supplementary material for: Ca2+-Dependent Glucose Transport in Skeletal Muscle by Diphlorethohydroxycarmalol, an Alga Phlorotannin: In Vitro and In Vivo Study
Source: Oxid Med Cell Longev. 2021 Feb 10;2021:8893679. doi: 10.1155/2021/8893679 (PMC7889350; doi:10.1155/2021/8893679)

*Supplementary protocol 1. Streptozotocin-induced diabetic mice and experimental design.* Twelve-week-old C57BL/6N male mice were used in this research. The mice were acclimated for 2 weeks. After acclimation, the mice injected intraperitoneally with STZ solution (60 mg/kg) in a citrate buffer (0.1 M, pH 4.5). After mice injected with STZ for 72 h, the mice were allocated into five treatment groups: Normal/Saline, Streptozotocin (STZ)/Saline, STZ/DPHC, STZ/Metformin, STZ/BAPTA+DPHC. Each group was orally administrated glucose (1 g/kg) or DPHC (30 mg/kg) or Metformin (100 mg/kg). In the muscle, to ensure if Ca^2+^ signal was blocked by chelation of cytosolic Ca^2+^ with BAPTA-AM, we pre-injected into mouse muscle with BAPTA-AM (2.5 mg/kg) for 30 min before glucose intake. And then, an intraperitoneal glucose tolerance test (IGTT) was performed and the data analyzed through the calculation of the area under curve (AUC). The mice experiment received approval from the Animal Care and Use Committee of the Jeju National University (Approval No. 2017-0001)

*Supplementary protocol 2. Western blot analysis.* The Glut4 were used at 1:1000 dilution, and secondary antibodies were used at 1:3000 dilution.

Figure S1. Evaluation of IGTT in streptozotocin-induced diabetic mice. (a) Measurement of blood glucose level after glucose intake in mice fed with 30 mg/kg DPHC or 100 mg/kg Metformin. Mice were injected with 2.5 mg/kg BAPTA-AM for 30 min, before glucose intake. (b) Quantitative analysis of the area under the curve (AUC) from IGTT. Data are expressed as the mean ± SE, *n* = 4 per group. ^*^ and ^#^ Values having different superscript are significantly different at ^*^*p*<0.05 compared with the no sample-treated group; ^##^*p*<0.01 compared with the non-treated group.

Figure S2. Expression of membrane and cytosolic Glut4 level in mice muscle tissues by western blotting. The muscle extract was analyzed by western bottling and the signal intensities were examined by the Fusion FX7 acquisition system (Vilbert Lourmat, Eberhardzell, Germany). Membrane Glut4 was normalized by cytosolic Glut4. Data are expressed as the mean ± SE, *n* = 4 per group. ^*^ and ^#^ Values having different superscript are significantly different at ^*^*p*<0.05, and ^***^*p*<0.001 compared with the no sample-treated group; ^###^*p*<0.001 compared with the non-treated group.

Figure S1.


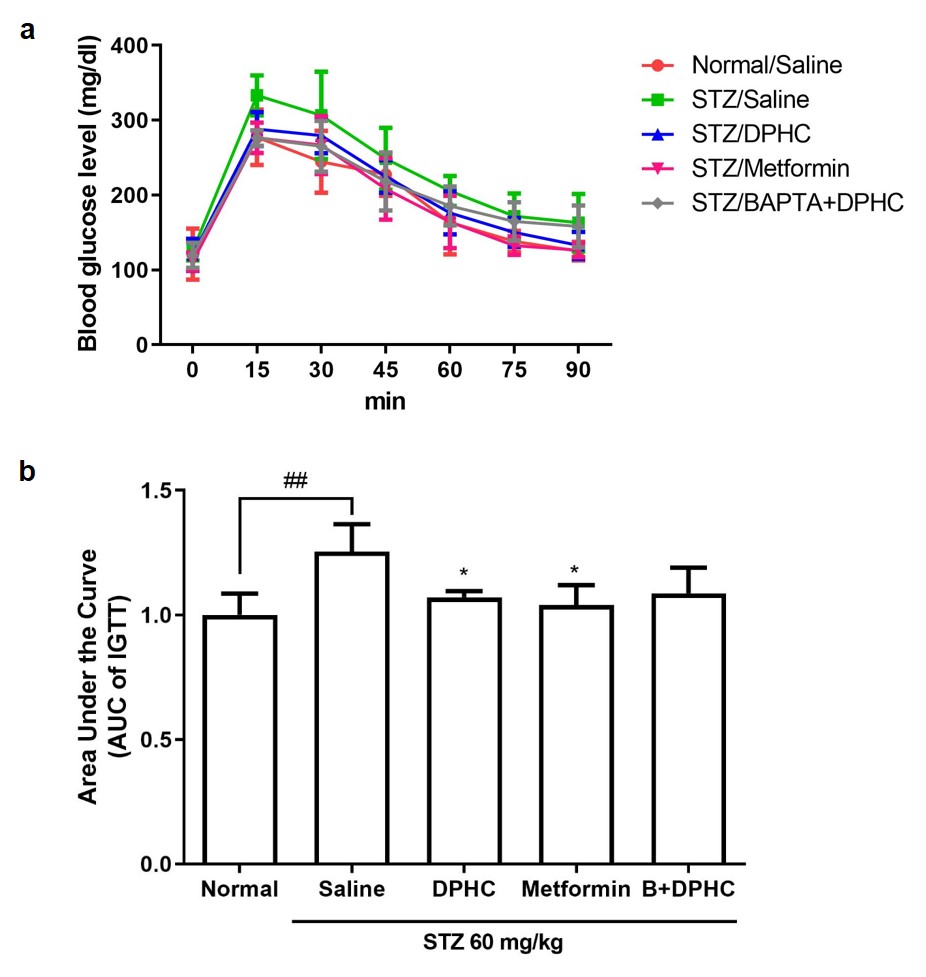


Figure S2.


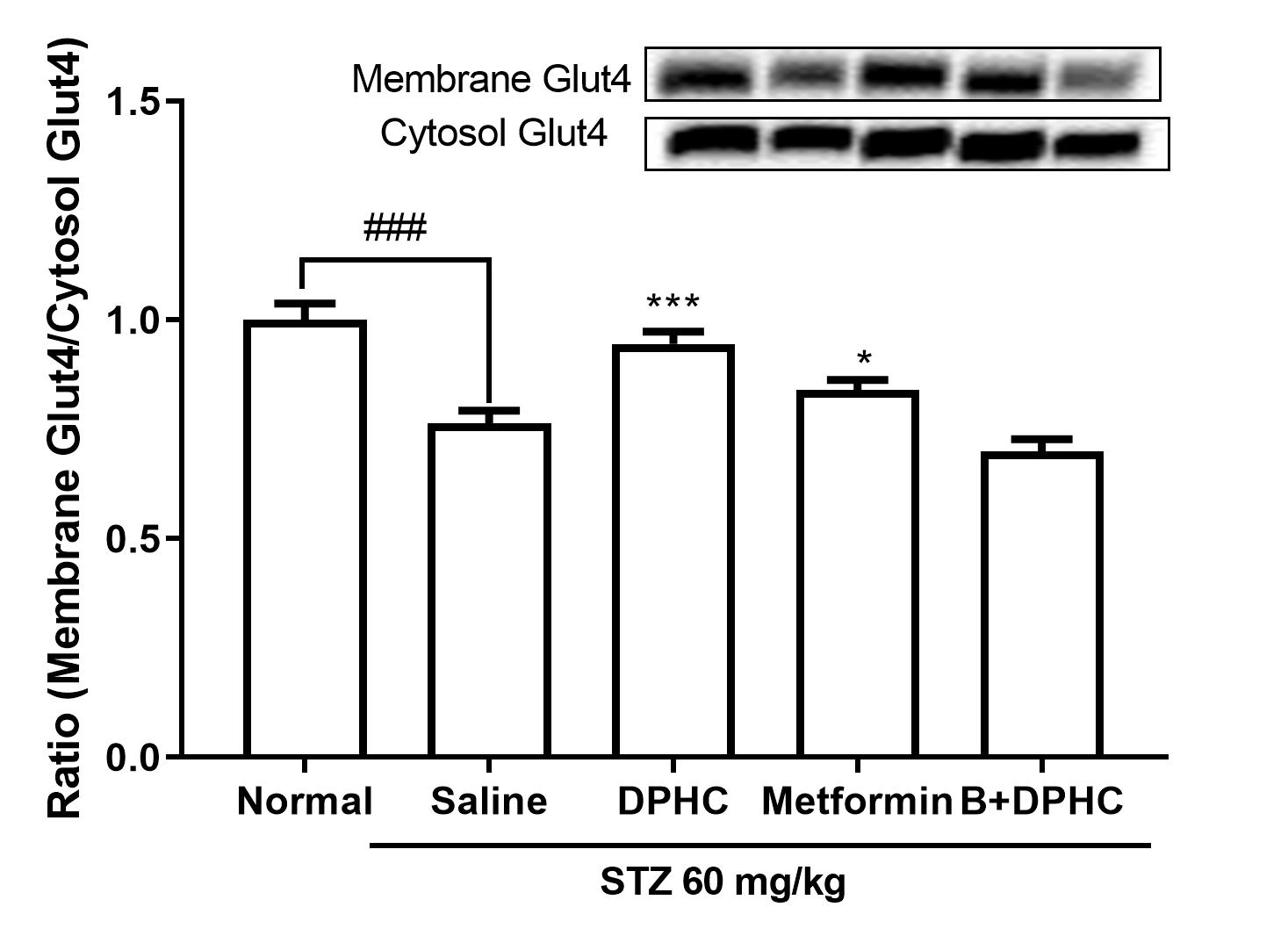

Supplement: Supplementary Materials — Figure S1: evaluation of IGTT in streptozotocin-induced diabetic mice. (a) Measurement of blood glucose level after glucose intake in mice fed with 30 mg/kg DPHC or 100 mg/kg metformin. Mice were injected with 2.5 mg/kg BAPTA-AM for 30 min, before glucose intake. (b) Quantitative analysis of the area under the curve (AUC) from IGTT. Data are expressed as the mean ± SE, n = 4 per group. ∗,#Values having different superscripts are significantly different at ∗p < 0.05 compared with the no sample-treated group; ##p < 0.01 compared with the nontreated group. Figure S2: expression of membrane and cytosolic Glut4 level in mice muscle tissues by western blotting. The muscle extract was analyzed by western bottling, and the signal intensities were examined by the Fusion FX7 acquisition system (Vilber Lourmat, Eberhardzell, Germany). Membrane Glut4 was normalized by cytosolic Glut4. Data are expressed as the mean ± SE, n = 4 per group. ∗,#Values having different superscripts are significantly different at ∗p < 0.05 and ∗∗∗p < 0.001 compared with the no sample-treated group; ###p < 0.001 compared with the nontreated group. [file 8893679.f1.docx]
